# Single-nucleotide polymorphisms in the *RB1* gene and association with breast cancer in the British population

**DOI:** 10.1038/sj.bjc.6603160

**Published:** 2006-05-09

**Authors:** F Lesueur, H Song, S Ahmed, C Luccarini, C Jordan, R Luben, D F Easton, A M Dunning, P D Pharoah, B A J Ponder

**Affiliations:** 1 Department of Oncology, University of Cambridge, Strangeways Research Laboratories, Cambridge CB1 8RN, UK; 2 Department of Public Health and Primary Care, University of Cambridge, Strangeways Research Laboratories, Cambridge CB1 8RN, UK; 3 Department of Genetic Epidemiology, University of Cambridge, Strangeways Research Laboratories, Cambridge CB1 8RN, UK

**Keywords:** *RB1*, single-nucleotide polymorphisms, breast cancer

## Abstract

A substantial proportion of the familial risk of breast cancer may be attributable to genetic variants each contributing a small effect. pRb controls the cell cycle and polymorphisms within it are candidates for such low penetrance susceptibility alleles, since the gene has been implicated in several human tumours, particularly breast cancer. The purpose of this study was to determine whether common variants in the *RB1* gene are associated with breast cancer risk. We assessed 15 tagging single-nucleotide polymorphisms (SNPs) using a case–control study design (*n*⩽4474 cases and *n*⩽4560 controls). A difference in genotype frequencies was found between cases and controls for rs2854344 in intron 17 (*P*-trend=0.007) and rs198580 in intron 19 (*P*-trend=0.018). Carrying the minor allele of these SNPs appears to confer a protective effect on breast cancer risk (odd ratio (OR)=0.86 (0.76–0.96) for rs2854344 and OR=0.80 (0.66–0.96) for rs198580). However, after adjusting for multiple testing these associations were borderline with an adjusted *P*-trend=0.068 for the most significant SNP (rs2854344). The *RB1* gene is not known to contain any coding SNPs with allele frequencies ⩾5% but several intronic variants are in perfect linkage disequilibrium with the associated SNPs. Replication studies are needed to confirm the associations with breast cancer.

Breast cancer is the most common cancer in women worldwide and women in the UK have a one out of 10 lifetime risk of developing the disease. First-degree female relatives of breast cancer patients have an approximately two-fold increased risk over the general population, but less than 25% of this excess risk is explained by inherited mutations in known high penetrance breast cancer susceptibility genes, such as *BRCA1* and *BRCA2* (Antoniou *et al*, 2001). Data from large multiple case families suggest that there will be few other high penetrance genes. It is more plausible that there are multiple common low risk (low penetrance) genetic variants, which are associated with relatively small effects on risk in the individual, but contribute substantially to the overall risk in the population (Antoniou *et al*, 2002).

Controlling the progression of cells into and through S phase of the cell cycle is important in regulating DNA synthesis and thus cell proliferation. The retinoblastoma protein (pRb) is critical for regulating not only progression of cells from G1 into S phase, but also progression of cells through S phase (Weinberg, 1995; Knudsen *et al*, 1998; Niculescu *et al*, 1998). It acts as a negative regulator of cellular proliferation by sequestering a variety of nuclear proteins involved in cellular growth that are released when pRb is phosphorylated. The gene *RB1*, encoding the protein, was mapped to chromosome 13q14.12–13q14.2 in children who developed retinoblastoma, a rare cancer of the eye, and was the first tumour-suppressor gene to be cloned (Friend *et al*, 1986). It consists of 27 exons that are distributed over 180 kb. Mutations are spread across the gene and approximately 80% of patients with hereditary mutations have bilateral disease. Hereditary retinoblastoma patients are at risk of developing and dying of second primary cancers in childhood and adolescence (Francois *et al*, 1980; Draper *et al*, 1986; Lueder *et al*, 1986; DerKinderen *et al*, 1988; Olsen *et al*, 1990) and excess mortality from second malignancies in retinoblastoma survivors was found to persist during long-term follow-up into adulthood. Female patients have a higher mortality from second tumours (RR=39) than males (RR=22) (Eng *et al*, 1993). Germline mutations in specific codons or regions of the *RB1* gene could therefore predispose to the development of a second tumour. Subsequent studies have shown somatic mutation of *RB1* in a variety of cancers, including sarcomas, breast cancer, lung cancer and genitourinary cancers (Benedict *et al*, 1988; T'Ang *et al*, 1988; Hensel *et al*, 1990; Sasano *et al*, 1990). Common variants in *RB1* are therefore candidate for low to moderate risk breast cancer alleles.

Association studies, using very large sets of affected cases and suitably selected controls, are considered to be the most powerful method for finding common low penetrance disease susceptibility genes. The aim of this study was to test the hypothesis that one or more variants in the gene is associated with breast cancer using a single-nucleotide polymorphism (SNP) tagging approach in a large, British breast cancer case–control study. In order to have good power to detect small relative risks we have restricted our attention to common SNPs and haplotypes (frequency ⩾5%).

## PATIENTS, MATERIALS AND METHODS

### Patients and controls

Cases were drawn from SEARCH (breast), an ongoing population based study, with cases ascertained through the East Anglian Cancer Registry. All patients diagnosed with invasive breast cancer below age 55 years since 1991 and still alive in 1996 (prevalent cases, median age 48 years), together with all those diagnosed <70 years between 1996 and the present (incident cases, median age 54 years) were eligible to take part. In all, 67% of eligible breast cancer patients returned a questionnaire and 64% provided a blood sample for DNA analysis. Controls were randomly selected from the Norfolk component of European Prospective Investigation of Cancer (EPIC). European Prospective Investigation of Cancer is a prospective study of diet and cancer being carried out in nine European countries. The EPIC-Norfolk cohort comprises 25 000 individuals resident in Norfolk, East Anglia) – the same region from which the cases have been recruited. Controls were not matched to cases, but were broadly similar in age, being aged 42–81 years old at blood draw (median age 63 years). The ethnic background of both cases and controls as reported on the questionnaires was similar, with >98% being white. The study was approved by the Eastern Region Multicentre Research Ethics Committee, and all patients gave written informed consent.

The total number of cases available for analysis was 4474 of whom 27% were prevalent cases. The samples have been split into two sets in order to save DNA and reduce genotyping costs: the first set (*n*=2271 cases and 2280 controls) is genotyped for all SNPs and the second set (*n*=2203 cases and 2280 controls) is then tested for those SNPs that show marginally significant associations in set 1 (*P*-heterogeneity or *P*-trend <0.1). This staged approach substantially reduces genotyping costs without significantly affecting statistical power. Cases were randomly selected for set 1 from the first 3500 recruited, with set 2 comprising the remainder of these plus the next 974 incident cases recruited. As the prevalent cases were recruited first, the proportion of prevalent cases was somewhat higher in set 1 than set 2 (33 *vs* 20%). Median age at diagnosis was similar in both sets (51 and 52 years old, respectively). There was no significant difference in the morphology, histopathological grade or clinical stage of the cases by set or by prevalent/incident status.

### Identification of SNPs

Single-nucleotide polymorphisms were initially identified through the following SNP databases: ENSEMBL, http://www.ensembl.org/, dbSNP, http://www.ncbi.nlm.nih.gov/SNP, and The *RB1* gene mutation database, http://www.d-lohmann.de/Rb/polym_t2.html (Lohmann, 1999). Eleven SNPs, encompassing the *RB1* gene and with a reported frequency ⩾5% in the Caucasian population according to the public databases were initially examined in a set of 96 individuals from the EPIC-Norfolk population in order to confirm their presence in the British population (Table 1) and to estimate pairwise correlation coefficients (*r*_p_^2^). Two SNPs, rs198610 and rs198580 were subsequently found to have a frequency lower than 5% in our East Anglian sample set. Strong linkage disequilibrium (LD) across the *RB1* gene was observed (illustrated by *r*^2^ values shown in Table 1).

During the course of the study the NIEHS EGP Project (http://pga.gs.washington.edu/finished_genes.html) released resequencing data for the coding sequence of *RB1* gene. This represents 38% of the genomic sequence. Data were available for a panel of 90 individuals representative of US ethnicities: including 24 European Americans, 24 African Americans, 12 Mexican Americans, 6 Native Americans and 24 Asian Americans (PDR90) (Livingston *et al*, 2004). It is known that there is greater genetic diversity in individuals of African origin but ethnic group identifiers for the PDR90 samples are not available. We identified 28 of the samples most likely to be African-American in this population by comparing the genotypes for the PDR90 samples with the genotypes for the same SNPs from the National Heart, Lung, and Blood Institute Variation Discovery Resource project African-American panel. Data from the remaining 62 individuals were used to identify a set of tagging SNPs (stSNPs). Of 279 SNPs identified in the PDR90 samples, only 25 are likely to have a frequency ⩾0.05 in Caucasians (Table 1).

We used the programme Tagger to select a set of SNPs to tag all the known common variants (Paul de Bakker, http://www.broad.mit.edu/mpg/tagger). Tagger uses a strategy that combines the simplicity of pairwise methods with the potential efficiency of multimarker approaches. It begins by selecting a minimal set of markers such that all alleles to be captured are correlated at an *r*_p_^2^ greater than 0.8 with a marker in that set. It then tries to capture SNPs which could not be captured in the pairwise step using multimarker tests constructed from the set of markers chosen as pairwise tags.

Four of the SNPs we had initially chosen were not present in the PDR90 data set (all in intron 17). The remaining seven were forced in as tagging SNPs. An additional eight tagging SNPs were chosen, but an assays could not be designed for two of these, neither of which had alternative tags. Another tSNP was found not to be polymorphic in our population. Thus, 12 tSNPs were genotyped in our case–control sample. These tagged 22 out of 25 SNPs with *r*_p_^2^>0.8 and one SNP (rs1573601) was tagged by a three SNP haplotype combination. The SNPs which failed assay design were tagged with *r*_p_^2^=0.19 and *r*_p_^2^=0.28. The contribution of the four additional SNPs identified through public databases to the tagging of other known variants could not be estimated.

### Genotyping

We genotyped all samples for the 15 SNPs using the ABI PRISM 7900 sequence detection system or ‘Taqman’ (Applied Biosystems, Foster City, CA, USA). Forward and reverse primers, and FAM and VIC labelled probes were designed by Applied Biosystems (ABI Assays-by-Design or ABI Assays-on-Demand). Sequences for primers and probes are available on request. We carried out PCR on DNA (10 ng) using TaqMan universal PCR master mix (Applied Biosystems) in a 5 *μ*l reaction. Amplification conditions on MJ Tetrad thermal cyclers (GRI) were as follows: one cycle of 95°C for 10 min, followed by 40 cycles of 95°C for 15 s and 60°C for 1 min. We read the completed PCRs on an ABI PRISM 7900 Sequence Detector in end point mode using the Allelic Discrimination Sequence Detector Software (Applied Biosystems). For the software to recognise the genotypes, we included two nontemplate controls in each 384-well plate. For set 1 and set 2, cases and controls were arrayed together in twelve 384-well plates and a thirteenth plate contained eight duplicate samples from each of the twelve plates to insure a good quality of genotyping. For each SNP, failed genotypes were not repeated.

### Statistical methods

For each polymorphism, deviation of the genotype frequencies from those expected under Hardy–Weinberg equilibrium was assessed in the controls by *χ*^2^ tests. Genotype frequencies in cases and controls were compared by *χ*^2^ tests (*P*-heterogeneity, 2 d.f.). We also tested for an allele dose effect assuming a multiplicative codominant model using unconditional logistic regression (*P*-trend, 1 d.f.). The genotypic specific risks were estimated as odds ratios (ORs) with associated 95% confidence limits. For SNPs that were significant at the 5% level we also compared the fit of dominant and recessive models with the codominant model by combining the appropriate genotype categories.

In addition to the univariate analyses we carried out global haplotype test and a specific haplotype test for a three SNP haplotype that tagged a common variant. Haplotype frequencies and subject-specific expected haplotype indicators were calculated separately using the programme TagSNPs, which implements an expectation-substitution approach to account for haplotype uncertainty given unphased genotype data (Stram *et al*, 2003a, 2003b). Subjects missing more than 50% genotype data were excluded from haplotype analysis. We considered haplotypes with greater than 4% frequency in either cases or controls to be ‘common’. Rare haplotypes were pooled. We used unconditional logistic regression to test the global null hypothesis of no association between haplotype frequency and breast cancer, by comparing a model with multiplicative effects for each common haplotype (treating the most common haplotype as referent) to the intercept-only model. Haplotype-specific ORs were also estimated with their associated confidence intervals.

### Screening of the *P2RY5* gene

We looked for the presence of polymorphisms with rare allele frequency ⩾5% in the *P2RY5* gene (RefSeq NT_024524) in our population by sequencing a set of 48 genomic DNA samples from the UK breast cancer patients. Sequencing was performed on the ABI Prism 3100 Capillary DNA Sequencer (Applied Biosystems) according to the manufacturer recommendations. The pairs of primers used for the sequencing of the *P2RY5* gene are available from authors on request.

## RESULTS

### Association analysis of SNPs

Genotype distributions in the controls did not differ significantly from those expected under Hardy–Weinberg equilibrium for any of the SNPs. Of the 15 SNPs, 12 were genotyped only in set 1. SNPs rs2854344 and rs4151611 were tested in set 2 because they met the threshold for significance (see methods) and rs198580 was also tested in set 2 because we had observed a borderline association for this SNP with another disease phenotype (data not shown).

There was no significant difference in genotype frequencies between cases and controls for SNPs rs1981434, rs2854345, rs520342, rs399413, rs4151540, rs198610, rs2227311, rs9535032, rs425834, rs4151611, rs4151620, rs3092904 and rs4151636 (Table 2). For two SNPs, rs2854344 and rs198580, unadjusted *P*-values for comparison of genotype distribution between cases and controls were below the 5% level (*P*-heterogeneity=0.02 and 0.03 and *P*-trend=0.007 and 0.018, respectively). Genotype-specific risks for all tagging SNPs are in Table 2. There was no association in controls between age and genotype frequency for any of the SNPs, and age-adjusted genotype-specific ORs were similar to the unadjusted ORs (data not shown). The minor allele of rs2854344 appeared to confer a reduced risk of disease with a dominant model fitting the data slightly better than the codominant one (*P*=0.006 *vs* 0.007). The dominant model also fit the data best for rs198580 (*P*=0.01 *vs* 0.02), also with a protective effect of the minor allele, even though the risk estimate for rare homozygous individuals was 1.4 (0.31–6.24).

One SNP, rs1573601, was tagged by the haplotype consisting of the three rare alleles of rs1981434, rs4151540 and rs3092904. The frequency of this haplotype was similar in cases (0.25) and in controls (0.25) (*P*=0.94).

### Haplotype analysis

As the complete gene was not resequenced, it is possible that important functional variants that have not been tagged by the 15 SNPs genotyped could have been missed. We therefore carried out a comparison of common haplotype frequencies in cases and controls in addition to the univariate and specific three SNP haplotype analyses. There were five common haplotypes which accounted for 84% of all haplotypes in the control population (Table 3). We found no evidence of differences in common haplotype frequencies between cases and controls (*P*=0.08, 5df). Table 3 shows the haplotype-specific ORs, none of which differed significantly from the unity.

### Exclusion of the *P2RY5* gene

The SNP rs2854344 lies in intron 17 of *RB1*, which at 72 kb is the largest intron of the gene. The intron contains an open reading frame encoding the G protein-coupled receptor *P2RY5* (Purinergic Receptor P2Y, G-protein coupled, 5) in the reverse orientation relative to the transcription of *RB1* (Herzog *et al*, 1996). The *P2RY5* gene consists of only one coding exon and rs2854344 lies 11kb 5′ of this exon. Various bioinformatic tools (NIX, Nucleotide Identify X software, http://www.hgmp.mrc.ac.uk/NIX; PupaSNP, http://pupasnp.bioinfo.cnio.es) suggest that the variant rs2854344 (and the variant rs198580 in intron 19 of *RB1*) does not have any functional effect, or alter dramatically the structure of *RB1* or of *P2RY5* (data not shown). Therefore, causal variant(s) in LD with rs2854344 and rs198580 could be located within *RB1* or within *P2RY5*. In order to investigate a possible association with variants within *P2RY5*, we sequenced the unique exon of *P2RY5* and 500 bp of its flanking sequences in 5′ and 3′ in a panel of 48 controls. No coding SNP was identified. The nearest polymorphisms were rs2227311, located 473 bp upstream, and rs4151551, located 86 bp downstream of *P2RY5.* No association with breast cancer risk was found with rs2227311 (Table 2), and rs4151551 was subsequently tested in set1. No association with breast cancer risk was found with rs4151551 (*P*-heterogeneity=0.74). We conclude that, if the observed associations are real, it is likely that it is variation related to *RB1* and not *P2RY5* that modifies breast cancer susceptibility.

## DISCUSSION

The case–control study design is well suited to the identification of small-effect genes that are likely to underlie common, complex diseases such as breast cancer (Risch, 2000). Two approaches have been proposed. The traditional, hypothesis-driven approach is to investigate SNPs on the basis of their putative biological relevance, in particular SNPs in coding regions, as they are more likely to influence directly the traits under study (Tabor *et al*, 2002). Alternatively, when many markers, both coding and noncoding, are available in a gene, it may be more efficient to select only tagging SNPs, that is, SNPs that capture the majority of the genetic variation of the gene (Johnson *et al*, 2001; Stram *et al*, 2003a, 2003b; Thompson *et al*, 2003). There are no common coding SNPs in the *RB1* gene and since the regulatory SNPs have not yet been characterised we chose the indirect approach, which allows detection of association between a particular genomic region and the disease, whether or not the SNPs themselves have a functional effect (Gabriel *et al*, 2002; Cardon and Abecasis, 2003; Zondervan and Cardon, 2004). We are confident that our set of selected markers provides enough information about the remainder of the common SNPs in the gene, and any unknown common variants will either be tagged by the stSNPs or by the common haplotypes that they generate (Haiman *et al*, 2003; The International HAPMAP Consortium, 2003; Carlson *et al*, 2004). It is worth noting that the two SNPs that were associated with breast cancer were not identified in the EGP resequencing data, and would have been missed if only these data had been used to select tSNPs.

We found that the minor alleles of two SNPs, rs2854344 and rs198580 were associated with breast cancer susceptibility at a nominal significance level of 0.05. However, we have tested 15 SNPs for association and the possibility that the findings are the result of a Type I statistical error should not be discounted. Standard adjustments for multiple hypothesis testing, such as the Bonferroni correction, are too conservative, as they assume that the tests are independent. We therefore used permutation testing by randomly shuffling the case–control status to obtain an empirical adjusted *P*-value for the most significant association detected in the primary tests of association (i.e. *P*-trend=0.007). In 1000 random permutations, a *P*-value at least as significant as this was obtained on 68 occasions, giving a *P*-trend adjusted for multiple testing of 0.068 for the association of rs2854344 with breast cancer. Thus, the observed association is of borderline significance.

An alternative explanation for the observed results is confounding due to hidden population stratification. This occurs when allele frequencies differ between population subgroups and cases and controls are drawn differentially from those subgroups. However, it seems unlikely that population stratification is relevant here because the cases and controls were drawn from the same ethnic groups (both >98% of northwestern European ancestry). Furthermore, we have found no evidence for association between pairs of 64 unlinked markers (2016 tests) in the controls, which suggests that there is unlikely to be significant substructure in our population (Goode *et al*, 2005).

Assuming the results to be real, it may either be due to a direct causative effect of the SNPs tested, or it may be because they are markers for other functional variants. The associated SNPs lie in intron 17 and intron 19 of *RB1*, and it seems unlikely that either of them has direct functional effects. pRb undergoes cell-cycle-dependent phosphorylation during G_1_, and this modifies its interaction with at least some members of the E2F family, which regulates the transcription of many genes required for S phase (Kaelin *et al*, 1992). The finding of protective *RB1* alleles was unexpected, as deletions or inactivating mutations of the gene observed in tumours generally lead to an absence of negative control of the protein on the cell proliferation. No coding variants were identified during EGP resequencing and so it is plausible, but unlikely that the presence of the still unidentified common variant prevents the protein from appropriate dephosphorylation. However, only a small number of subjects were resequenced and it is possible that the observed association is due to correlation with an unidentified rare coding variant that was not present in the resequenced samples. Nor have we been able to identify any common variants in *P2RY5*, the gene within intron 17 of *RB1* that might explain the association. Again rare variants cannot be excluded. The more likely hypothesis is the presence of a SNP in the promoter or in a regulatory element that affects the level of pRb. It is possible that the causal variant might induce higher levels of pRb or expression at critical times than the common allele.

Further studies would be required to identify and investigate the mechanism of action of a causative variant. However, before such studies are contemplated, these putative associations need to be tested and confirmed in independent breast cancer studies. It would also be interesting to check the involvement of the protective alleles in other cancer types, in particular in melanoma, and in bone, connective tissue, ovarian and uterine cancer which are also cause of death in retinoblastoma long term survivors (Eng *et al*, 1993).

## Figures and Tables

**Table 1:** Selection of SNPs across *RB1*

**SNP ID**	**Genomic location**	**Nucleotide change^a^**	**SNPs chosen initially for genotyping**	**SNP reported by NIEHS with MAF>0.05**	**Tag SNP**	**Tagging SNP(s)**	***r*_p_^2^**
rs1573601	Upstream	c>a		✓		rs1981434, rs4151540, rs3092904 (g/+/a haplotype)	0.93
rs1981434	Intron 1	c>g		✓	✓	rs1981434	1.0
rs2854345	Intron 2	a>g	✓	✓	✓	rs2854345	1.0
rs4151437	Intron 2	g>a		✓		rs4151551	0.80
rs4151438	Intron 2	c>g		✓	FAIL^b^	rs2854345	0.19
rs520342	Intron 3	c>t	✓	✓	✓	rs520342	1.0
rs4151450	Intron 3	g>c		✓		rs399413	0.85
rs198619	Intron 7	t>a		✓		rs399413	0.84
rs4151510	Intron 11	g>a		✓		rs4151620	1.0
rs4151520	Intron 11	g>a		✓		rs520342	0.96
rs399413	Intron 12	g>a	✓	✓	✓	rs399413	1.0
rs4151540	Intron 17	->aa		✓	✓	rs4151540	1.0
rs198610	Intron 17	g>t	✓		?^c^	?	
rs4151551	Intron 17	g>t	✓	✓		rs4151551	1.0
rs2227311	Intron 17	t>c	✓	✓	✓	rs4151620	1.0
rs2854344	Intron 17	g>a	✓		?^c^	?	
rs9535032	Intron 17	a>g	✓		?^c^	?	
rs425834	Intron 17	a>g		✓	✓	rs425834	1.0
rs1951775	Intron 17	g>t		✓		rs399413	0.85
rs198570	Intron 17	g>t		✓		rs3092904	1.0
rs4151580	Intron 18	g>a		✓	Not polym^d^	rs4151580	1.0
rs4151584	Intron 18	t>c		✓	FAIL^b^	rs2854345	0.28
rs198580	Intron 19	a>g	✓		?^c^	?	
rs198590	Intron 21	g>a		✓		rs520342	1.0
rs4151611	Intron 24	g>a		✓	✓	rs4151611	1.0
rs4151618	Intron 24	t>c		✓		rs4151611	1.0
rs4151620	Intron 24	c>g	✓	✓	✓	rs4151620	1.0
rs3092904	Intron 24	t>a	✓	✓	✓	rs3520342	0.98
rs4151636	Downstream	c>g		✓	✓	rs4151636	1.0

a The most common allele is given first.

b The SNP was initially selected for genotyping but the assay could not be designed.

c The contribution of the SNP to the tagging of other identified SNP could not be estimated.

d The SNP was not polymorphic in the British population.

**Table 2:** SNPs genotyped in the study set

**SNP**	**Series**	**Minor allele frequency**	**Common homozygote *n* (%)^a^**	**Heterozygote *n* (%)^a^**	**Rare homozygote *n* (%)^a^**	**Number genotyped**	***P*-trend**	***P*-het**
rs1981434	Cases	0.28	1114 (51)	866 (40)	191 (9)	2171		
	Controls	0.29	1171 (52)	905 (40)	188 (8)	2264	0.66	0.84
	OR (95% CI)		1 (ref)	1.01 (0.89–1.14)	1.07 (0.86–1.33)			
rs2854345	Cases	0.19	1309 (65)	631 (31)	66 (3)	2006		
	Controls	0.18	1489 (68)	625 (29)	77 (3)	2191	0.15	0.12
	OR (95% CI)		1 (ref)	1.15 (1.01–1.31)	0.98 (0.70–1.37)			
rs520342	Cases	0.25	1121 (56)	759 (38)	126 (6)	2006		
	Controls	0.25	1232 (57)	793 (36)	149 (7)	2174	0.93	0.56
	OR (95% CI)		1 (ref)	1.05 (0.93–1.20)	0.93 (0.72–1.19)			
rs399413	Cases	0.29	1032 (51)	819 (41)	164 (8)	2015		
	Controls	0.27	1157 (53)	855 (39)	164 (8)	2176	0.20	0.42
	OR (95% CI)		1 (ref)	1.07 (0.95–1.22)	1.12 (0.89–1.41)			
rs4151540	Cases	0.28	1144 (53)	865 (40)	168 (8)	2177		
	Controls	0.27	1220 (54)	874 (38)	179 (8)	2273	0.63	0.68
	OR (95% CI)		1 (ref)	1.06 (0.93–1.19)	1.00 (0.80–1.25)			
rs198610	Cases	0.03	1893 (93)	131 (6)	3 (<1)	2027		
	Controls	0.03	2081 (94)	129 (6)	3 (<1)	2213	0.43	0.69
	OR (95% CI)		1 (ref)	1.12 (0.87–1.43)	1.10 (0.22–5.45)			
rs2227311	Cases	0.13	1548 (76)	452 (22)	41 (2)	2041		
	Controls	0.14	1645 (74)	521 (24)	49 (2)	2215	0.25	0.49
	OR (95% CI)		1 (ref)	0.92 (0.80–1.06)	0.89 (0.58–1.35)			
rs2854344	Cases	0.07	3634 (87)	542 (13)	23 (1)	4199		
	Controls	0.08	3738 (84)	659 (15)	29 (1)	4426	0.007	0.023
	OR (95% CI)		1 (ref)	0.80 (0.75–0.96)	0.82 (0.47–1.41)			
rs9535032	Cases	0.29	1020 (50)	830 (41)	180 (9)	2030		
	Controls	0.29	1134 (51)	884 (40)	189 (9)	2207	0.48	0.76
	OR (95% CI)		1 (ref)	1.04 (0.92–1.19)	1.06 (0.85–1.32)			
rs425834	Cases	0.03	2052 (94)	135 (6)	5 (<1)	2192		
	Controls	0.03	2138 (94)	135 (6)	3 (<1)	2276	0.62	0.71
	OR (95% CI)		1 (ref)	1.04 (0.81–1.33)	1.74 (0.41–7.28)			
rs198580	Cases	0.02	4001 (95)	186 (4)	4 (<1)	4191		
	Controls	0.03	4187 (94)	251 (6)	3 (<1)	4441	0.018	0.033
	OR (95% CI)		1 (ref)	0.78 (0.64–0.94)	1.40 (0.31–6.24)			
rs4151611	Cases	0.05	3955 (91)	391 (9)	8 (<1)	4354		
	Controls	0.04	4160 (91)	387 (8)	7 (<1)	4554	0.38	0.68
	OR (95% CI)		1 (ref)	1.06 (0.92–1.23)	1.20 (0.44–3.32)			
rs4151620	Cases	0.13	1537 (76)	450 (22)	39 (2)	2026		
	Controls	0.14	1628 (75)	510 (23)	39 (2)	2177	0.55	0.62
	OR (95% CI)		1 (ref)	0.93 (0.81–1.08)	1.06 (0.67–1.66)			
rs3092904	Cases	0.26	1118 (55)	785 (38)	139 (7)	2042		
	Controls	0.26	1227 (56)	822 (37)	154 (7)	2203	0.71	0.75
	OR (95% CI)		1 (ref)	1.05 (0.92–1.19)	0.99 (0.78–1.26)			
rs4151636	Cases	0.05	1996 (91)	187 (9)	5 (<1)	2188		
	Controls	0.04	2092 (92)	181 (8)	4 (<1)	2277	0.45	0.71
	OR (95% CI)		1 (ref)	1.08 (0.87–1.34)	1.31 (0.35–4.89)			

a Rounded to the nearest unit.

OR, odds ratio; CI, confidence interval.

**Table 3:** *RB1* haplotype analysis using the 15 SNPs genotyped in the study set

	**Frequency**					
**Haplotype^a^**	**Controls**	**Cases**	**OR (95% CI) compared to most common**	***P*-value**	**OR (95% CI) compared to all others**	***P*-value**
000000000000000	0.38	0.37	Ref	—	0.94 (0.86–1.03)	0.19
111110000100010	0.16	0.17	1.12 (0.98–1.27)	0.09	1.09 (0.97–1.22)	0.16
000000010000000	0.13	0.13	0.99 (0.86–1.13)	0.86	0.94 (0.83–1.07)	0.33
000000100000000	0.10	0.09	1.01 (0.86–1.18)	0.92	0.97 (0.83–1.12)	0.64
101110001100010	0.07	0.06	0.94 (0.78–1.12)	0.47	0.90 (0.75–1.06)	0.21
Rare^b^	0.07	0.08	1.16 (1.03–1.32)	0.02	1.15 (1.02–1.28)	0.02

a SNPs used for haplotype analysis have same order as Table 2. For each SNP, 0 represents the commonest allele and 1 the rarest allele.

b Rare haplotypes (⩽4%) were pooled.
